# Pathogenic gene screening in 91 Chinese patients with short stature of unknown etiology with a targeted next-generation sequencing panel

**DOI:** 10.1186/s12881-018-0730-6

**Published:** 2018-12-12

**Authors:** Lulu Yang, Chenhui Zhang, Wei Wang, Junqi Wang, Yuan Xiao, Wenli Lu, Xiaoyu Ma, Lifen Chen, Jihong Ni, Defen Wang, Jinxiu Shi, Zhiya Dong

**Affiliations:** 1 Department of Pediatrics, Ruijin Hospital affiliated to Shanghai Jiao Tong University School of Medicine, Ruijin 2nd Road 197, Shanghai, 200025 China; 2 Department of Genetics, Shanghai-MOST Key Laboratory of Health and Disease Genomics, Chinese National Human Genome Center and Shanghai Industrial Technology Institute (SITI), Keyuan Road 1278, Shanghai, 201203 China

**Keywords:** Short stature, Unknown etiology, Next generation sequencing (NGS), Targeted panel, Pathogenic genes

## Abstract

**Background:**

Dwarfism is a common severe growth disorder, but the etiology is unclear in the majority of cases. Recombinant human growth hormone may be a treatment option, but it has limited efficacy. The currently known laboratory assays do not meet the precision requirements for clinical diagnosis. Here, we have constructed a targeted next-generation sequencing (NGS) panel of selected genes that are suspected to be associated with dwarfism for genetic screening.

**Methods:**

Genetic screening of 91 children with short stature of unknown etiology was performed with the help of the NGS panel. All the coding regions and exon-intron boundaries of 166 genes were included in the panel. To clarify the pathogenicity of these mutations, their clinical data were reviewed and analyzed.

**Results:**

The assay identified p.A72G, p.I282V, and p.P491S variants of the *PTPN11* gene and a p.I437T variant of the *SOS1* gene in 4 cases with Noonan syndrome. A frameshift mutation (p.D2407fs) of the *ACAN* gene was identified in a case of idiopathic short stature with moderately advanced bone age. A p.R904C variant of the *COL2A1* gene was found in a patient, who was accordingly diagnosed with Stickler syndrome. Severe short stature without limb deformity was associated with a p.G11A variant of *HOXD13*. In addition, we evaluated evidence that a p.D401N variant of the *COMP* gene may cause multiple epiphyseal dysplasia.

**Conclusions:**

Our findings suggest that syndromes, particularly Noonan syndrome, may be overlooked due to atypical clinical features. This gene panel has been verified to be effective for the rapid screening of genetic etiologies associated with short stature and for guiding precision medicine-based clinical management.

## Background

Short stature is a common pediatric endocrine disease with an incidence of 3–5% [1]. Most of the cases have an unclear etiology, which makes drug selection and prognostic counseling difficult. Growth is a complex process that involves many genes and is affected by heredity, hormones, nutrition, and the environment. Heritability factors accounting for 80% of human height outcomes are suspected to be the main causes of individual variations in growth. Endocrine and paracrine factors associated with intracellular signaling pathways of the growth plate could improve our understanding of bone growth. With the advancement of molecular technologies, more genetic testing methods for studying human stature have been developed, but there is still a need for clinically acceptable tests [2].

In this study, an online database and the relevant literatures were searched for candidate genes associated with dwarfism, including genes associated with the hypothalamic-pituitary IGF-1 axis and the bone growth plate, especially endocrine and paracrine growth factors and intracellular signaling pathways. A total of 166 genes were identified (Table 1). A gene testing panel containing all the coding regions and exon-intron boundaries of these genes was developed in house. Finally, the gene variation data were analyzed in combination with clinical phenotype data to determine the likely molecular etiologic diagnosis when possible.

**Table 1:** List of 166 genes in gene panel

GH/IGF axis	Growth hormone deficiency	POU1F1 PROP1 LHX3 LHX4 HESX1 OTX2 PITX1 SOX2 SOX3 SPR
		GLI2 GLI3 IGSF1 FGF8 FGFR1 PROKR2 HMGA2 GPR161
		GHRHR GH1BTK GHSR ALMS1 RNPC3 IFT172
	GH insensitivity	GHR STAT5B STAT3 IL2RG IGF1 IGF2 IGFALS PAPPA2 IKBKB
	IGF insensitivity	IGF1R
paracrine factors in the growth plate	FGF signaling	FGFR2 FGFR3
	BMP signaling	GDF5 BMPR1B BMP2
	WNT signaling	ROR2 WNT5A
	PTHrP-IHH pathway	PTHLH PTH1R IHH GNAS PRKAR1A PDE4D
	CNP-NPR2 pathway	NPR2
cartilage extracellular matrix		FBN1 PAPSS2 IDUA COL10A1 COL9A1 COL9A2 COL9A3 SLC26A2 COMP COL2A1 ACAN ADAMTS10
intracellular pathways	SHOX aberrations	SHOX
	Rasopathies	PTPN11 SOS1 KRAS NRAS HRAS RAF1 BRAF RIT1 NF1 RPS6KA3
	Other syndromes	FGD1 SOX9 KMT2D KDM6A TBCE FAM111A
fundamental cellular processes	Syndrome	CHD7 SEMA3E SMARCB1 SMARCA2 ARID1A ARID1B SRCAP ANKRD11 TRIM37 PIK3R1 CUL7 OBSL1 CCDC8 NIPBL SMC1A SMC3 RAD21 CDT1 CDC6 PCNT TUBGCP6 MECP2 CREBBP EP300 ATR CENPJ CEP152 CEP63 DNA2 ATRIP CRIPT
	DNA repair defects	WRN BLM ERCC8 ERCC6 ERCC3 ERCC5 ERCC4 LMNA PCNA SMARCAL1 MCM4 MCM9 NBN RECQL4 ATRX LIG4 NHEJ1 ARTEMIS (DCLRE1C) XRCC4 FANCA
Other		THRB THRA TSHR TG FOXE1 IYD DUOX2 SLC5A5 ESR1 VDR DHCR7 CYP19A1 INSR PITX2 CDK6 DRD2 IGFBP3 JAK2 HOXD13 MMP13 NOG RUNX2 TRPV4 FGF2 NPPC NPR3 COL1A1 SHOX2 MAPK1 NFKB1 AKT1 AKT2 GRB2 GRB10 ZBTB38 CDKN1C EHD1

## Methods

### Materials

From July 2007 to December 2015, 91 children with short stature who were being followed at Ruijin Hospital Affiliated to Shanghai Jiaotong University School of Medicine were enrolled in our cohort for pathogenic screening. They met the following criteria for short stature: body height lower than minus two standard deviation units (-2SDS) as compared to their normal same-age/same-sex peers [3]. Exclusion criteria included the following: history of being small for gestational age, growth hormone deficiency (defined as growth hormone peak value < 10 ng/ml on the arginine and clonidine stimulation test), hypothyroidism, chronic organic diseases, known chromosomal aberrations or inborn errors associated with metabolic diseases, and growth failure secondary to psychological factors. Informed written consent was obtained from participant or guardians. In our study, 90 cases were under the age of 16; for these participants, their guardian provided written informed consent. In addition, one case of 16.7 years provided written informed consent. Ethical approval was provided by the Ruijin Hospital Ethics Committee Shanghai JiaoTong University School of Medicine.

The documented medical history included the status of their birth, feeding, growth, development and past illness as well as of the family members. Physical examination was used to measure body height, weight, head circumference, sitting height, arm span and signs of sexual development. Laboratory issues evaluated the results of growth hormone provocative test, levels of IGF-1 [4]. Radiographic imaging was used to assess bone age, by the method of Greulich–Pyle Atlas. All statistical analyses were conducted using SPSS 16.

### Next-generation sequencing

Genomic DNA was isolated from peripheral lymphocytes using FlexiGene DNA Kit(Qiagne) and quantified using a NanoDrop ND2000 (NanoDrop Technologies, Wilmington, DE, USA) spectrophotometer. A total of 1000 ng of each DNA sample was fragmented on a Bioruptor Plus sonication system (Diagenode, Belgium). Sheared DNA was used to perform end repair, A-tailing and adapter ligation with the KAPA LTP Library Preparation kit according to the manufacturer’s protocol. Then, 750 ng of prepared DNA in a volume of 3.4 μl was captured using in-house developed RNA baits; this was followed by amplification of the captured library by indexing primers. After qualitative analysis with the Agilent 2100 bioanalyzer (Agilent Technologies) on the DNA chip and quantitative analysis with the Qubit® 3.0 fluorometer (Invitrogen), the libraries were sequenced on an Illumina platform (Illumina Inc., San Diego, CA, USA).

RNA baits were generated from an oligo pool synthesized by Azco Biotech. The oligo pool was amplified into double-stranded DNA. Then, a T7 promoter site was incorporated into the amplicon, and the DNA was transcribed into biotinylated RNA. The biotinylated RNA was purified, quantified, and prepared for target enrichment.

Variant calling and coverage analysis of each captured region were performed using an in-house developed bioinformatics pipeline based on the general analysis pipeline. Briefly, high-quality reads were mapped to the hg19 version of the human reference genome (GRCh37) using the BWA aligner with the BWA-MEM algorithm and default parameters. The Genome Analysis Toolkit was used to locally realign the BAM files at intervals with indel mismatched and recalibrate base quality scores of reads in the BAM files. Variants were identified by the GATK HaplotypeCaller and annotated by annovar. The variants and annotation results were transferred into Excel spreadsheets.

All the variants with MAF greater than 0.01 in ExAc or 1000G were filtered. To analyze these variants, we referred dbSNP, CliVar, Exac, and HGMD database and screened out significative variations with the Variant Effect Predictor, MutationTaster, MutationAssessor,PolyPhen2, SIFT, and other predictors of mutation effects on protein function combined with the ACMG evaluation guide [5]. These variants of pathogenic, likely pathogenic and uncertain significance were verified by Sanger sequencing. Finally, to clarify the likely molecular etiologic diagnosis, the gene variation data were analyzed in combination with clinical phenotype data.

## Results

### General data

A total of 91 participants (56 males and 35 females) with an average age of 9.3 ± 3.2 years (range, 2.0–16.7 y) were enrolled in this study. The average height standard deviation score (Ht-SDS) of patients was − 2.84 ± 0.63 SD (range, − 2.02–-5.45 SD). The target height for the boys was 166.3 ± 4.1 cm, and for the girls, it was 154.2 ± 3.5 cm. There were three with cryptorchidism, one with a cleft palate, three with atrial septal defects, and one with hearing loss.

### Genetic variants identified

A pathogenic or probable pathogenic variant was identified in 8 of the 91 patients. The clinical data of these 8 cases are shown in Table 2. The group included four patients with Noonan syndrome, three with heterozygous mutations of the *PTPN11* gene, and one with an *SOS1* mutation. In addition, one patient with a mildly advanced bone age was found to harbor a frameshift mutation of the *ACAN* gene; whereas, one patient with early-onset myopia, cleft palate, flat nose, and short fingers was found to have a hemizygous mutation of the *COL2A1* gene. Furthermore, our findings suggest that p.G11A of *HOXD13* may cause severe short stature without limb deformity, and that the p.D401N variant of the *COMP* gene may cause multiple epiphyseal dysplasia. The identified mutations are shown in Table 3.

**Table 2:** Clinical data of patients with genetic variants identified with the gene screening panel

ID	Age range at initial appointment (years)	Ht-SDS	CA-BA (years)	GH peak (ng/ml)	IGF-1 (ng/ml)	IGF-SDS	Father’s ht. (cm)	Mother’s ht. (cm)	Clinical description
P1	11~13	-5.45	3.9	10.0	113	−2.58	165.0	158.0	Unilateral hearing loss, atrial septal defect, cryptorchidism, wide-spaced nipples, hypertelorism, language delay, family history remarkable for brother who died of congenital heart disease
P2	4~6	−4.18	1.0	11.1	60(50–286)		168.5	149.0	Facial anomalies (a high, arched palate and flat nose), atrial septal defect
P3	4~6	−2.51	1.7	17.2	101	−1.36	178.0	153.0	Facial anomalies (small chin, neck skin webbing, epicanthal folds, gothic arch), atrial septal defect, cryptorchidism, language delay, learning disabilities, sternal deformities (pectus carinatum superiorly)
P4	7~9	−4.08	4.0	12.0	76	−1.74	176.0	161.0	Wide-spaced eyes, low nasal bridge, short neck
P5	11~13	−2.36	−0.4	10.0	521	−0.02	170.0	141.0	Menarche at 12 years
P6	4~6	−2.94	1.3	18.3	240(49–283)		170.0	156.0	Early-onset myopia, cleft palate, flat nose, short fingers
P7	4~6	−4.33	3.0	29.4	33(50–286)		167.0	148.0	No obvious characteristics
P8	7~9	−2.83	4.5	19.0	104	−2.05	163.0	146.0	Short limbs (height 117 cm, arm span 110 cm); mild gait disturbance; short bone hand deformity and mild lumbar scoliosis on imaging (as shown in Fig. 1). His father was also short and presented with similar bone deformity with short and limp legs (height 163 cm, arm span 153 cm), in addition to bilateral avascular necrosis of the femoral head on imaging (as shown in Fig. 2).

*M* male, *F* Female

**Table 3:** Genetic variants identified

ID	Gene	Transcript	cDNA	Protein	Exon	Inheritance	Origin	Zygosity	Variant/ACMG
P1	*PTPN11*	NM_002834	c.215C > G	A72G	E3	AD	–	Het	P/PS1 PS3 PM1 PM2 PP2 PP3
P2	*PTPN11*	NM_002834	c.844A > G	I282V	E7	AD	de novo	Het	P/PS1 PS2 PS3 PM1 PM2 PP2 PP4
P3	*PTPN11*	NM_002834	c.1471C > T	P491S	E13	AD	de novo	Het	P/PS1 PS2 PS3 PM1 PM2 PP2 PP4
P4	*SOS1*	NM_005633	c.1310 T > C	I437T	E10	AD	de novo	Het	P/PS1 PS2 PS3 PM1 PM2 PP2 PP4
P5	*ACAN*	NM_013227	c.7222dupA	D2407fs	E16	AR,AD	–	Het	P/PVS1 PM2 PM4 PP3 PP4
P6	*COL2A1*	NM_001844	c.2710C > T	R904C	E41	AD	–	Het	P/PS1 PM1 PM2 PP2 PP3
P7	*HOXD13*	NM_000523	c.32G > C	G11A	E1	AD	–	Het	LP/PS3 PM2 PP3
P8	*COMP*	NM_000095	c.1201G > A	D401N	E11	AD	Father	Het	LP/PM1 PM2 PP2 PP3 PP4

*AD* Autosomal dominant inheritance, *AR* Autosomal recessive inheritance, *PVS* very strong pathogenic likelihood, *PS* strong pathogenic likelihood, *PM* moderate pathogenic likelihood, *PP* pathogenic supporting, *P* pathogenic, *LP* likely pathogenic

## Discussion

Short stature is one of the most common conditions encountered by pediatric endocrinologists. Adult human height is influenced by factors varying across heredity, hormonal, nutrition, and environmental factors. Growth is a complex process regulated by many genetic factors, and numerous monogenic causes of growth disorders have been identified. Endocrine and paracrine factors are involved in the growth of long bones, and chondrocyte proliferation, hypertrophy and secretion of cartilage extracellular matrix are known to also be essential to growth [6, 7].

This study identified 166 growth-related genes (involving the endocrine system, paracrine growth factors, extracellular matrix, and intracellular signaling molecules in the growth plate) as candidates to construct a panel for screening genetic defects associated with short stature. From our cohort of 91 children with short stature of unknown etiology, 8 pathogenic mutations were identified.

Three children with atrial septal defects (P1–P3) were found to carry heterozygous variants (p.A72G, p.I282V, and p.P491S) of the *PTPN11* gene, of which the p.I282V and p.491S variants were found in the parental samples, too, and are believed to be de novo mutations. These three variants have been reported to be associated with Noonan syndrome in other populations [8–10], but there are no reports about these variants in China. The *PTPN11* gene encodes SHP-2, the widely expressed cytoplasmic protein tyrosine phosphatase. As a downstream molecule of growth factor and cytokine receptors, the SHP-2 signal molecule participates in the RAS-MAPK signaling pathway, which is involved in organ development, hematopoiesis and metabolism, as well as cell proliferation, differentiation, and migration. *PTPN11* gene defects interfere with the RAS-MAPK signaling pathway, resulting in damage to multiple systems related to organ development. SHP-2 consists of tandemly arranged N-SH2 and C-SH2 domains and a PTP catalytic domain [11]. Studies have shown that most of the pathogenic variations are located within the N-SH2 and PTP domains [12]. In the case of the three genetic variants identified here, related studies have revealed that the 3 variants lead to inactivation of SHP-2, instability of conformation, and enhancement of SHP-2 function, resulting in the occurrence of Noonan syndrome [8–10]. Noonan syndrome is autosomal dominant and characterized by postnatal growth retardation [13], specific facial features, congenital heart defects, and skeletal deformities [14–16]. In our three cases of Noonan syndrome, all three patients were heterozygous for the variant and showed similar phenotypes: congenital heart disease (P1, P2, P3), facial feature anomalies (P1, P2, P3), short stature (severe in P1 and P2 and mild in P3), cryptorchidism (P1 and P3) and language delay (P1 and P3).

In this study, we also found a de novo heterozygous mutation (*SOS1*:p.I437T) in P4, who displayed severe short stature (− 4.08 SD), hypertelorism, a flat nose, and a short neck. The *SOS1* gene encodes guanine nucleotide exchange factor, which is responsible for the transformation of Ras from its inactive GDP binding state to its active GTP binding form. After activation, an MAPK cascade reaction occurs due to Ras activating downstream MEK1/2 and ERK1/2, resulting in the activation of multiple nuclear transcription factors and kinases in the nucleus and, thereby, affecting gene transcription and regulating cell function. As a large multi-domain protein, SOS1 consists of an N-terminal regulatory part, a Dbl homology domain, a pleckstrin homology domain, and a C-terminal catalytic region. Noonan syndrome has been reported to be associated with changes in the pleckstrin homology domain structure due to I437L variations [16]. With vastly heterogeneous clinical and genetic features, Noonan syndrome involves multiple systems and multiple organs. Early detection of this syndrome can help to identify the involved systems as soon as possible, to start early treatment, and to avoid misdiagnosis or a missed diagnosis.

Our patient P5 was diagnosed with idiopathic short stature (− 2.36 SD) with mildly advanced bone age (half a year) and without any other abnormalities; this patient carried a new variant, c.7222dupA (p.D2407fs), of the *ACAN* gene, which encodes the proteoglycan core protein (aggrecan). This protein is mainly expressed in articular cartilage and cartilage growth plates. Recent studies have found that heterozygous defects (e.g. p.L2355P) in the CLD domain can lead to a rare autosomal dominant disease characterized by symmetric short stature, advanced bone age, and premature growth arrest [17]. The p.D2407fs is a newly discovered mutation located in exon 16, a highly conserved region of CLD. This frameshift mutation results in premature termination of codons, insufficient haploid doses, and impaired protein function. Unfortunately, we were unable to verify the inheritance pattern of this mutation, although her mother was also short (height, 141 cm). Therefore, it is suggested that clinicians pay attention to this genetic defect (short stature with advanced bone age) when excluding known genetic causes of dwarfism.

P6 carried the known pathogenic variant p.R904C of *COL2A1* and was diagnosed with Stickler syndrome by gene screening. The Type II collagen protein encoded by *COL2A1*, the main source of collagen synthesis in chondrocytes, contains 1487 amino acids and has a three-helix domain extension consisting of a long uninterrupted glycine-X-Y chain. The R904C mutation resulted in the substitution of cysteine for arginine at the X position in the three-helix glycine-X-Y repeated sequence. Existing research shows that this substitution produces collagen molecules, which interfere with cartilage matrix protein interactions, secretion, and extracellular assembly into fibrils [18]. As result of abnormal secretion of collagen, extracellular fibrils cannot correctly recombine, and this interferes with endochondral ossification and linear bone growth. The p.R904C gene mutation has been reported to lead to collagen tissue diseases, which are characteristic of Stickler syndrome [18–21]. Stickler syndrome is a group of clinically and genetically heterogeneous collagen tissue diseases that involve the eyes, mouth, face, inner ear, bones, and joints. The classic phenotype includes symptoms of early-onset severe myopia, hearing impairment, and bone damage. Our patient P6 (− 2.94 SD) was found to have early-onset myopia, midface hypoplasia (flat nose, a high, arched palate), and skeletal involvement (brachydactyly). Although symptoms may be nonspecific, a pathogenetic diagnosis can be made by gene panel screening.

Patient P7 in our cohort carried the p.G11A mutation of the *HOXD13* gene (member of the HOX gene family). *HOXD13* is one of the major genes that determine the development of extremities as well of the vertebrae and limbs. *HOXD13* is located in 2q31 and contains 2 exons. The encoded protein consists of 335 amino acids. In this mutation, the 32nd guanine in the coding region (c.32G > C) of the gene’s first exon is substituted with cytosine, and the 11th amino acid of the HOXD13 protein is changed from glycine to alanine, without alteration in amino acid polarity and pH. This mutation has been reported to cause a SPD phenotype, and misexpression of *HOXD13*(p.G11A) in the developing chick phenocopies the human SPD phenotype [22]. SPD is characterized by a connection between the middle and ring fingers and fourth and fifth toes, variably associated with postaxial polydactyly in the same digits. In the case of P7, we are unable to explain the absence of limb deformity or the lack of correlation between the phenotype and genotype. Further studies are needed to clarify the findings in this case.

**Fig. 1:**
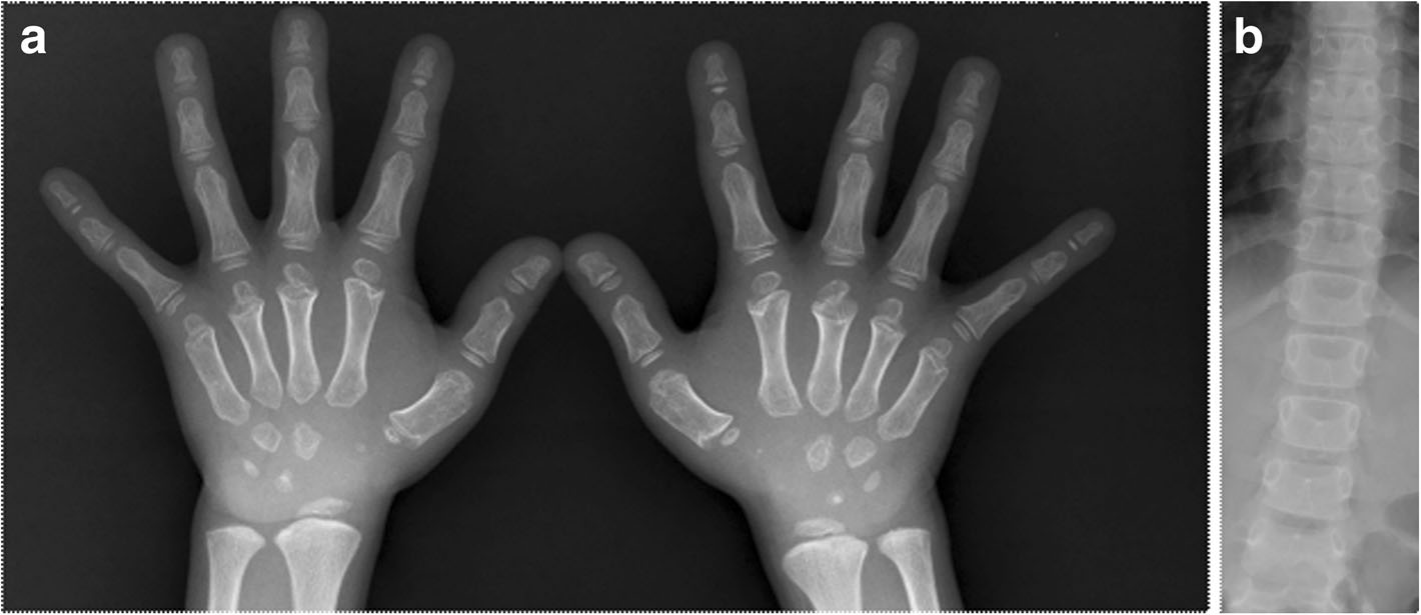
**a**: Short bone deformity of both hands, delayed development of osteophytes. **b**: Mild lateral curvature of the lumbar spine

**Fig. 2:**
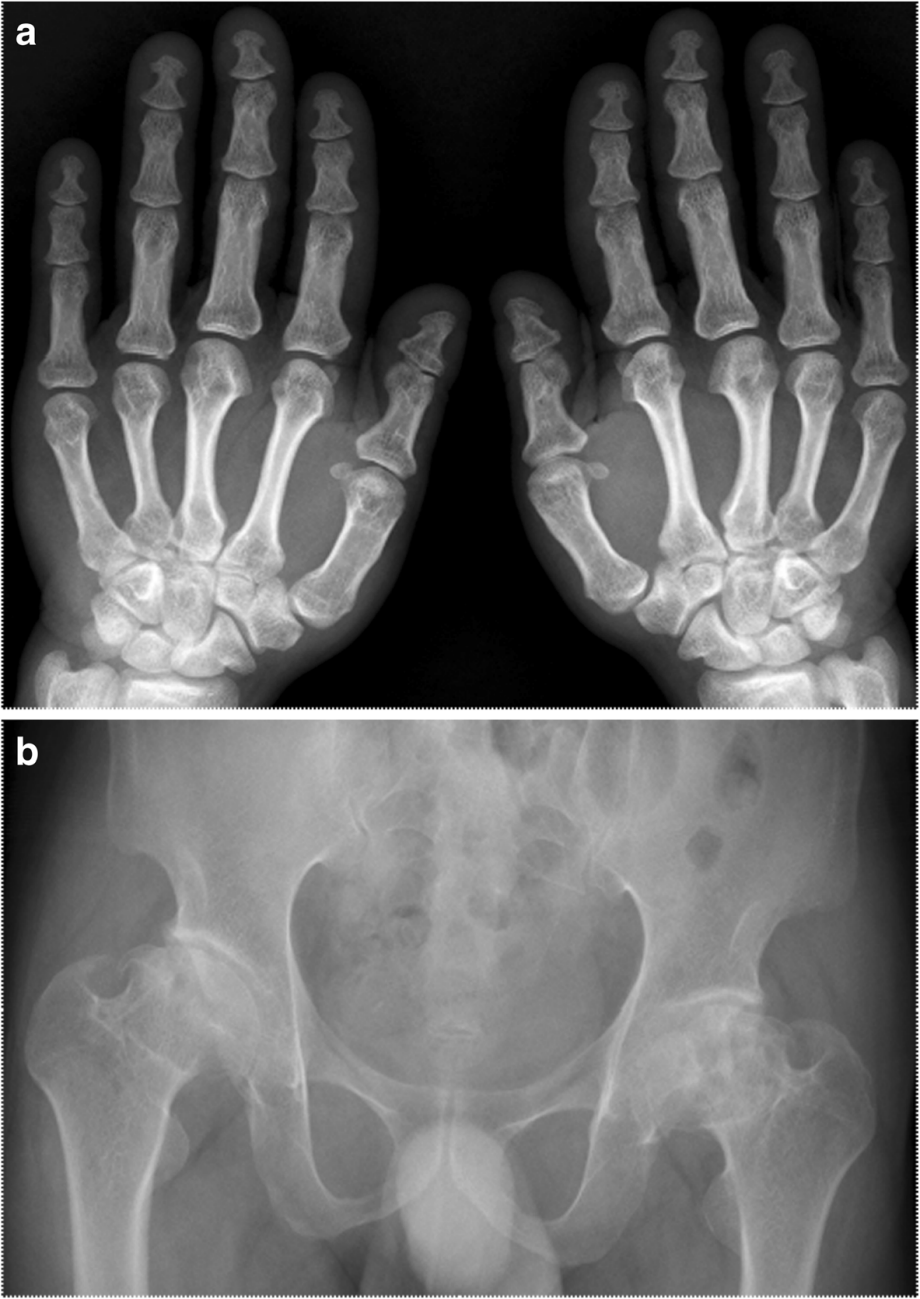
**a**: Short bone deformity of both hands. **b**: Bilateral avascular necrosis of the femoral head

In this study, P8 presented with familial short stature and carried a heterozygous variation of c.1201G > A within the *COMP* gene that resulted in the conversion of 401 aspartic acid into asparagine (p.D401N). *COMP* encodes an oligomeric matrix protein, a homopentameric glycoprotein primarily expressed in cartilage, tendons, ligaments, and skeletal muscle. Previous studies have shown that most of the pathogenic variants of *COMP* are located in the CLRs region [23, 24], which is encoded by exons 8–14. The variations in this pathogenicity region lead to protein misfolding and abnormal COMP deposition in the rough endoplasmic reticulum of chondrocytes; this results in chondrocyte endoplasmic reticulum stress and an unfolded protein response, apoptosis, and bone linear growth effects [25–27]. The D401N variant found in P8 and his father is located in the CLRs region encoded by exon 11, in the mutation hot spot. Heterozygous defects in *COMP* may result in multiple epiphyseal dysplasia [28], which is associated with mild growth disorders, hip and knee joint pain, swaying gait, and irregular epiphyseal ossification on imaging. Our patient displayed mild short stature (− 2.59 SD), delayed bone age (CA of 8.58 y and BA of 4.58 y), and short bone hand deformity and mild lumbar scoliosis on imaging (as shown in Fig. 1). Similarly, with short bone deformity, his father also had an abnormal pelvis radiograph that showed bilateral avascular necrosis of the femoral head (as shown in Fig. 2). The genetic defect associated with this gene new variant was also identified by gene panel screening, but the mechanisms underlying its genotype and phenotype remain to be clarified. Therefore, more functional experimental research is required to confirm its pathogenicity.

## Conclusions

In summary, a specific gene panel was developed based on molecular regulation mechanisms of linear bone growth to screen for clinical variants related to unexplained dwarfism. Through the use of this screening panel in 91 children with unexplained short stature, gene mutations were confirmed in 8 cases, including four cases with Noonan syndrome, one case with Stickler syndrome, a case of advanced bone age, a case of severe short stature without limb deformity, and a case of multiple epiphyseal dysplasia. Our findings suggest that some known causes of congenital bone disease and syndromes (such as multiple epiphyseal dysplasia and Noonan and Stickler syndromes) may be associated with atypical clinical features and be easily overlooked. Such cases are often attributed to idiopathic short stature. However, gene screening can be used for a more accurate diagnosis in some dwarf patients with unknown etiologies, and diagnosis may help guide the rational treatment of such patients. In this study, screening with the NGS panel was used to identify the genetic etiology of some patients with short stature and provided a precision science basis for clinical phenotype classification and genetic counseling. In addition, gene screening may provide the basis for the further establishment of effective interventions and help to meet the requirements for precision medicine. Further functional experimental research on variants of probable pathogenic significance is required.

## Abbreviations

ACMG: American College of Medical Genetics and Genomics Institute
BA: Bone age
CA: Chronological age
CLD: C-type lectin domain
CLRs: Calmodulin-like(type III)repeats
GH: Growth hormone
HGMD: Human gene mutation database
Ht-SDS: Height- Standard deviation score
IGF-1: Insulin-like growth factor-1
MAF: Minor allele frequency
MAPK: Mitogen-activated protein kinase
NGS: Next-generation sequencing
PTP: Protein tyrosine phosphatase
SDS: Standard deviation score
SHP: Tyrosine phosphatase
SPD: Synpolydactyly
